# Digital Stress: Insights from Bibliometric, Scientometric, Meta-Analytic and Thematic Analyses

**DOI:** 10.3390/healthcare14060823

**Published:** 2026-03-23

**Authors:** Ahmed Yahya Almakrob, Ahmed Alduais

**Affiliations:** 1 Department of English Language and Literature, College of Sciences and Humanities, Prince Sattam bin Abdulaziz University, Al-Kharj 16278, Saudi Arabia; a.almakrob@psau.edu.sa; 2 Department of Psychology, Norwegian University of Science and Technology, 7034 Trondheim, Norway

**Keywords:** digital stress, technostress, bibliometric analysis, scientometrics, meta-analysis, digital well-being, Digital Stress Scale

## Abstract

Digital stress, the psychological strain from constant connectivity, is a growing challenge, but the research field remains conceptually fragmented. This study aims to (1) map the evolution of digital stress research via bibliometric and scientometric analyses; (2) quantify measurement consistency through a meta-analysis of the Digital Stress Scale (DSS); and (3) synthesize thematic trends to clarify the construct’s boundaries. A multi-method review was conducted, integrating bibliometric analysis of 215 documents (Scopus/WoS), Google Ngram analysis, a random-effects meta-analysis of 10 DSS studies (n = 8572), and a thematic analysis of keyword co-occurrence. Bibliometrics and Ngram analysis show the field is maturing, with publications rising sharply post-2020, distinguishing it from ‘technostress.’ The construct evolved from biomedical/engineering uses to a psychosocial concept linked to ‘social media’ and ‘mental health.’ The meta-analysis found a moderate pooled mean stress level (2.45 on a 1–5 scale, 95% CI: 2.12–2.78), falling within the ‘average’ range of U.S. norms. High heterogeneity (I^2^ = 99.7%) confirmed that cultural and contextual factors significantly moderate stress levels. Thematic analysis identified four key dimensions: conceptual ambiguity, contextual moderators, the digital transformation paradox, and digital well-being. Digital stress is a distinct, multidimensional construct encompassing social-evaluative pressures beyond original technostress models. This review consolidates its theoretical boundaries and confirms the DSS’s psychometric consistency, highlighting digital stress as a critical, context-dependent factor in human adaptation to technology.

## 1. Introduction

### 1.1. Purpose and Rationale

The accelerating digitalization of daily life has created novel psychosocial challenges across educational and professional settings. One emerging concern is digital stress, defined as psychological strain resulting from constant connectivity, information pressure, and technology-mediated demands [1,2]. Research in occupational [3,4] and academic contexts [5] links these demands to fatigue, burnout, and diminished well-being, establishing digital stress as a crucial focus for applied and theoretical inquiry [6].

Despite growing attention, conceptual fragmentation persists. Earlier models such as technostress emphasized work-related ICT strain [7], whereas recent work frames digital stress as a broader, socially embedded construct involving cognitive overload, emotional exhaustion, and social comparison [1,8,9]. The coexistence of overlapping terms—digital overload, online vigilance, digital fatigue—has produced varied operationalizations and inconsistent measures. Consequently, the literature lacks theoretical consolidation. For the purposes of this review, digital stress is defined as the psychological strain arising from socio-digital demands—including connectivity pressure, social-evaluative expectations, and information overload—experienced across everyday digital environments. This definition distinguishes digital stress from technostress, which focuses narrowly on work-related ICT demands, and from clinical stress constructs, treating digital stress as a psychosocial continuum relevant to occupational, academic, and leisure contexts. This review’s primary scope is the psychosocial conceptualization of digital stress. Non-psychosocial uses of the term (e.g., biomedical or engineering applications) are retained in the bibliometric corpus to accurately map the construct’s full disciplinary trajectory but are not substantively interpreted in the thematic or meta-analytic phases. Integrating bibliometric mapping, meta-analytic pooling, and thematic synthesis in a single study reduces definitional drift by jointly identifying where the construct’s boundaries are stabilizing and where measurement consistency holds across cultural contexts versus where contextual variability dominates.

This gap underscores the need for systematic synthesis. While bibliometric and scientometric approaches can map constructs such as technostress and digital well-being [10,11], no study has integrated bibliometric, scientometric, and meta-analytic methods to examine digital stress comprehensively. The present study therefore aims to: (1) map the evolution and structure of digital stress research; (2) conduct a meta-analysis of studies using the DSS [1] and its adaptations; and (3) synthesize conceptual and thematic insights that refine definitions and guide future research. The project contributes theoretically by distinguishing digital stress from related constructs, methodologically by integrating quantitative mapping and synthesis, and practically by informing evidence-based application of digital stress instruments.

### 1.2. Evolution of the Concept

Technostress originated in the 1980s to describe strain associated with workplace ICT demands, later organized into five creators: techno-overload, techno-invasion, techno-complexity, techno-insecurity, and techno-uncertainty. These reliably predict impaired well-being and work attitudes, with recent validations refining their measurement [12,13,14,15]. The concept of digital stress broadened this focus from workplace ICT to everyday, mobile, and social-media contexts, emphasizing pressures of connectivity and evaluation—captured by availability stress, approval anxiety, fear of missing out, connection overload, and online vigilance—particularly salient among adolescents and students [16,17,18,19].

Technostress emphasizes technology-driven job demands, whereas digital stress extends to socio-digital environments where platform norms and peer expectations function as stressors alongside ICT overload [12,16,19]. Both are framed by transactional stress theory and the Job Demands–Resources (JD-R) model, which differentiates stressors from responses: strain occurs when demands exceed coping resources. Empirical work links ICT demands to psychological fatigue and burnout, with physiological correlates in healthcare and corporate samples [20,21,22]. Mechanistically, role overload and information-processing load explain how digitalization precipitates exhaustion, whereas autonomy, social support, and digital leadership buffer these effects [23,24,25,26].

Technostress creators overlap with digital stress components, yet the latter uniquely integrates social-evaluative pressures under continuous connectivity. Instruments grounded in creators assess stressors, while newer tools measure affective–cognitive responses. Translations of the Digital Stressors Scale emphasize stressor exposure, whereas media-focused studies target approval anxiety, fear of missing out, and vigilance as intervention priorities. Organizational strategies—technical support, digital literacy, and leadership—complement individual coping and skills-building programs that reduce problematic technology use [27,28,29,30,31].

Although technostress and digital stress share theoretical roots in transactional stress theory and the JD-R model, they are best understood as related but distinct constructs rather than as nested or interchangeable concepts. Technostress is primarily work-centric, operationalized through five ICT-specific creators (overload, invasion, complexity, insecurity, uncertainty) that predict job-related strain [7,12]. Digital stress, by contrast, extends to everyday socio-digital environments—including social media, mobile communication, and peer-mediated online interaction—and incorporates dimensions of social evaluation and approval anxiety that fall outside the scope of classic technostress models [1,8]. Theoretically, the emergence of digital stress as a standalone construct is justified by (1) its multi-domain applicability across occupational, academic, and leisure contexts; (2) its inclusion of social-affective dimensions absent from technostress inventories; and (3) the development of dedicated psychometric tools, notably the Multidimensional Digital Stress Scale (MDSS; [1]), which captures unique variance beyond general ICT strain measures. This conceptual boundary, while recognized in the literature [9,16], requires consistent theoretical grounding to prevent further definitional drift.

### 1.3. Theoretical Models and Frameworks

Within ICT-related stress research, the JD-R model remains the predominant framework. It defines demands as effortful aspects of work that incur psychological or physiological costs, and resources as elements that reduce demands, facilitate goals, or promote growth [32,33]. JD-R structures much technostress evidence, specifying overload and invasion as demands and autonomy or digital support as resources [12,34]. Resources often buffer the effects of digital demands on well-being; for instance, organizational support moderates the impact of banking transformation stressors on performance [25]. JD-R is frequently paired with the challenge–hindrance distinction, explaining why some ICT demands foster engagement when appraised as opportunities but cause strain when appraised as constraints [35]. Applications beyond office settings—especially in remote work—affirm JD-R’s relevance for linking digital resources to engagement and mental health [36,37].

The Transactional Model of Stress and Coping complements JD-R by elucidating appraisal and coping processes: primary appraisal of threat or challenge and secondary appraisal of coping options [38]. Combining JD-R’s structural focus with transactional processes enhances explanations of digital strain, clarifying how individual appraisals mediate stress–outcome relations [34]. For instance, in customer-facing digital roles, appraisal tendencies and perceived demandingness shape how requests influence strain [39]. Demand–Control models highlight autonomy as a moderator of workload stress [40], while Person–Environment fit frameworks view misalignment between ICT demands and personal capacities as proximal stressors [41]. Despite conceptual diversity, reviews emphasize the value of JD-R and transactional perspectives as complementary frameworks for studying digitally intensified work [12,34].

### 1.4. Measuring Digital Stress

Measurement approaches distinguish stress responses from stressor exposure. The MDSS captures five components—availability stress, approval anxiety, fear of missing out, connection overload, and online vigilance—with strong internal consistency across studies [1,18,19]. Cross-cultural validations confirm its factor structure in Chinese and Norwegian samples [5,27]. In parallel, the Technostress Creators Inventory operationalizes five ICT demands functioning as stressors; its structure has been validated in occupational samples using higher-order models [13,14]. While self-report tools are efficient and comparable, they are susceptible to shared-method bias, highlighting the need for multimethod designs [42,43]. It is important to note that the meta-analysis reported in this review specifically synthesizes mean DSS scores across samples to assess cross-cultural level consistency—that is, whether comparable levels of digital stress are observed internationally. It does not pool reliability coefficients or factor-analytic fit indices, which would require a separate psychometric meta-analysis.

Objective and physiological indicators now complement questionnaires. Digital behaviour traces and usage analytics index real-time connection loads, enriching self-reported stress data [44]. Physiological and biological markers—for instance, cortisol or heart-rate variability—are increasingly combined with environmental assessments to capture psychophysiological responses to interruptions and multitasking, improving ecological validity [43,45]. The field is converging on a portfolio approach integrating subjective, objective, and biological indicators: MDSS for stress responses, technostress inventories for ICT demands, and physiological data for real-world validation [1,13,27].

## 2. Methods

### 2.1. Design

This review adopted an integrative multi-method design combining bibliometric mapping, meta-analytic synthesis, and thematic analysis to provide a comprehensive understanding of the digital stress literature. The bibliometric and scientometric components offered a quantitative overview of publication trends, intellectual structures, and emerging research fronts, following established science-mapping practices [10,11]. The meta-analysis provided statistical aggregation of psychometric findings from studies employing the DSS [1], thereby quantifying measurement reliability and identifying cross-cultural consistencies. Complementing these quantitative insights, the thematic synthesis enabled an interpretive exploration of conceptual meanings, theoretical orientations, and contextual nuances [46]. This triangulated design was selected to balance scope and depth—bibliometric analysis for breadth, meta-analysis for empirical robustness, and thematic synthesis for interpretive richness—reflecting recommendations for comprehensive knowledge integration in contemporary systematic reviews [47,48]. No artificial intelligence tools were used for article screening, selection, or analysis. All analytical procedures were conducted using the software and platforms specified in Section 2.2, Section 2.3 and Section 2.4: Scopus and Web of Science for database searches, bibliometrix/Biblioshiny (R) for bibliometric and scientometric analyses, MetaAnalysisOnline.com for meta-analytic computations, and manual author judgment for thematic curation.

### 2.2. Bibliometric & Scientometric Analysis Phase

The bibliometric and scientometric components of this study aimed to map the structure, growth, and thematic development of the research on digital stress. The Scopus search string was TITLE(“digital stress”) OR TITLE-ABS-KEY(“digital stress”), and the WoS string was TI=(“digital stress”) OR TS=(“digital stress”). Both searches were conducted in October 2025, with no language restrictions applied. All document types available in each database were included; grey literature and dissertations were excluded as they are not indexed in Scopus or WoS. Deduplication was performed using the merge function in the bibliometrix R package 5.0, retaining the record with fuller metadata when duplicates were identified. Data were retrieved from two major indexing databases—Scopus and Web of Science (WoS)—to ensure comprehensive coverage across disciplines and publication types. The WoS search retrieved 164 documents published between 1981 and 31 October 2025, while Scopus yielded 194 documents spanning 1987 to 31 October 2025. The search was first limited to titles containing the exact term “digital stress” and was subsequently expanded to topic and field searches to capture works where the construct appeared in abstracts or keywords. All available document types were included, encompassing journal articles, books, conference papers, and reviews. The two databases were then merged and cleaned for duplicates. After harmonization, 215 unique records were retained for final analysis, representing the most complete and non-redundant dataset on the topic to date.

The search strategy was intentionally restricted to the term ‘digital stress’ and its direct variants in order to trace the specific conceptual emergence, disciplinary migration, and bibliometric trajectory of this labeled construct. Broader keyword sets encompassing related terms (e.g., ‘online stress,’ ‘technostress,’ ‘digital fatigue,’ ‘internet stress’) were deliberately excluded at the primary retrieval stage, as their inclusion would conflate the developmental history of digital stress with the wider ICT-stress literature. This focused approach follows recommendations for construct-specific bibliometric mapping [11] and is consistent with prior science-mapping studies targeting emerging constructs [12]. We acknowledge, however, that this strategy may underestimate co-citation networks and cross-construct influence; future studies employing expanded Boolean queries across related terms would complement the present mapping.

**Figure 1:**
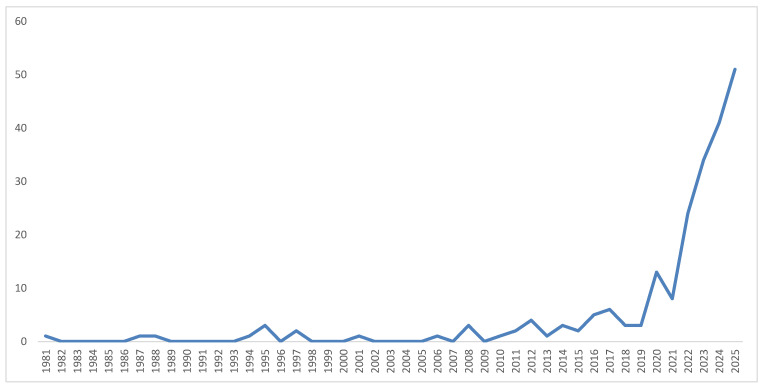
Annual Scientific Production on Digital Stress (1981–2025). *Note*. Note that country counts are based on full counting of author affiliations as generated by bibliometrix; a single document with multiple German-affiliated authors contributes multiple counts to Germany’s total. These figures therefore reflect affiliation-level scientific activity rather than unique document counts per country.

**Figure 2:**
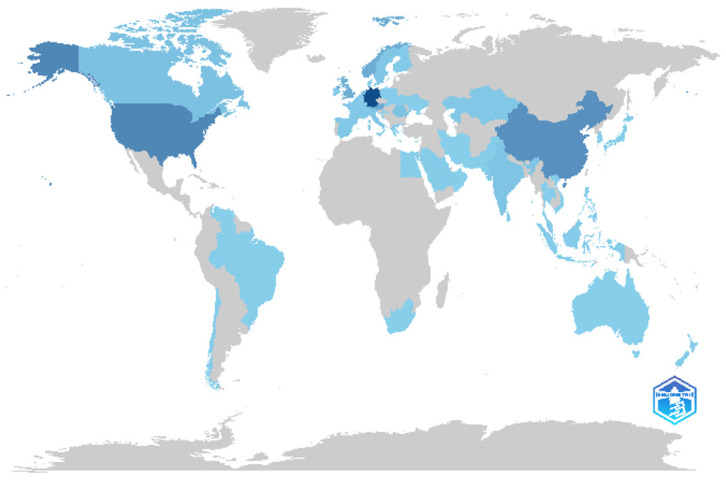
Country Scientific Production on Digital Stress Research. *Note*. Darker shades on the map represent higher scientific output on digital stress, while lighter shades indicate lower publication frequencies. Grey areas denote countries with no indexed publications in the dataset.

**Figure 3:**
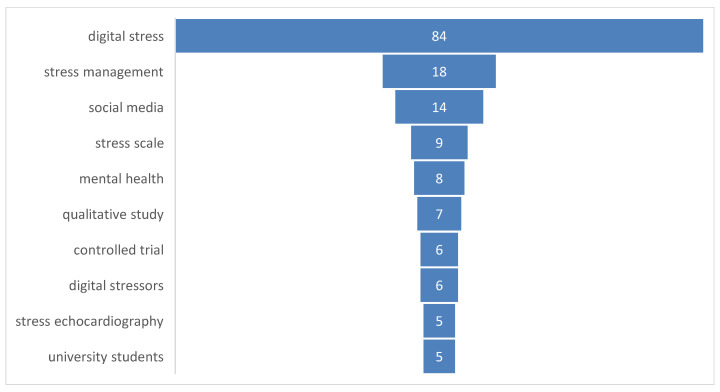
Most Frequent Author Keywords in Digital Stress Research.

**Figure 4:**
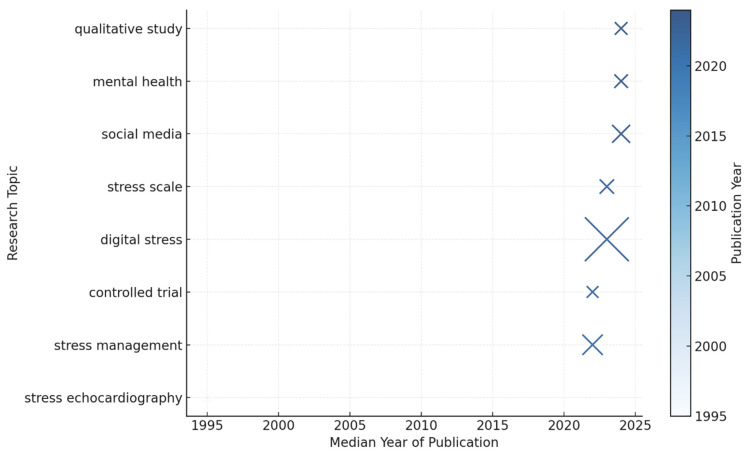
Emerging Research Topics and Temporal Distribution in Digital Stress Studies.

**Figure 5:**
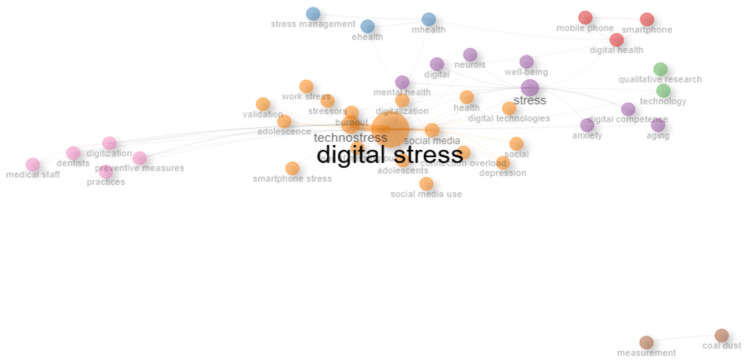
Co-Word Network of Digital Stress Research. *Note.* Network construction parameters: keywords appearing fewer than two times were excluded; co-occurrence counts were normalized using the association strength measure as implemented in bibliometrix. No algorithmic stemming or synonym merging was applied; minor manual harmonization (e.g., merging plural/singular variants) was performed during data cleaning. *Note*. In co-word network analysis, betweenness centrality indicates how strongly a keyword connects different thematic clusters—higher values mean a term serves as a conceptual bridge between research areas. PageRank, adapted from network theory, reflects a term’s overall importance within the network based on the number and strength of its links to other frequently occurring keywords. Keywords with high betweenness and PageRank, such as “digital stress,” are therefore central in shaping and linking the main research themes.

**Figure 6:**
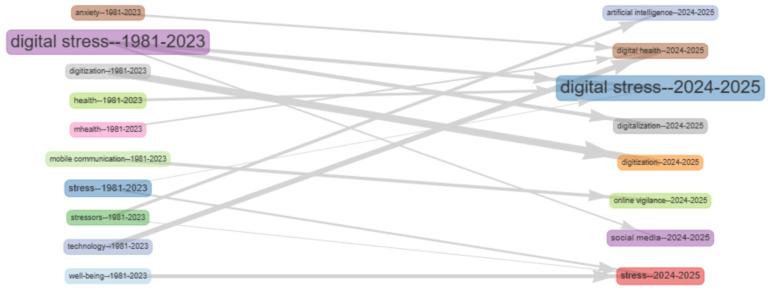
Thematic Evolution of Digital Stress Research (1981–2025).

All bibliometric and scientometric analyses were conducted using Biblioshiny (RStudio, version 4.3.3), an R-based application designed for bibliometric computation and visualization. Duplicate removal and data merging were performed using the latest bibliometrix package tools integrated within RStudio. The final corpus was analysed through descriptive and network-based measures, including document growth rates, authorship and collaboration patterns, country-level scientific production, and keyword co-occurrence. The analyses also included higher-order scientometric indicators such as co-word networks, thematic evolution, and keyword frequency analysis. Each dimension of analysis corresponds to one of Figure 1, Figure 2, Figure 3, Figure 4, Figure 5 and Figure 6 presented in the results section, representing annual scientific production, geographical distribution, author keyword frequency, emerging research topics, co-word networks, and thematic evolution.

### 2.3. Meta-Analysis Phase: Studies Using the Digital Stress Scale

To synthesize quantitative evidence from studies that reported descriptive statistics on the DSS [1], a random-effects meta-analysis was performed using summary-level data (mean, standard deviation, and sample size) extracted from each eligible study. The meta-analysis relied exclusively on publicly reported summary-level data (means, standard deviations, and sample sizes) extracted from published articles; no access to original participant-level datasets was sought or obtained. This aggregated approach is standard practice in single-group mean meta-analyses and is appropriate for quantifying cross-study level consistency; however, it precludes individual participant data analyses that could more precisely model moderating effects. Eleven published studies were identified, of which ten provided sufficient descriptive data for analysis. Because some studies reported DSS total scores (sum of all items) while others reported item-level means on a 1–5 Likert scale, all total-score values were standardized to an item-level metric to ensure comparability. This rescaling was performed by dividing the reported total mean and standard deviation by the number of items included in that study’s DSS version (ranging from 17 to 31 items). The standardized dataset therefore reflected the average level of digital stress per item (1 = very low to 5 = very high).

A random-effects model using the DerSimonian–Laird estimator was applied to account for between-study heterogeneity. In MetaAnalysisOnline.com, the following settings were applied: random-effects model with DerSimonian–Laird τ^2^ estimator, Knapp–Hartung adjustment for confidence intervals, and two-tailed tests at α = 0.05. The complete extracted dataset—including study identifiers, reported means, standard deviations, sample sizes, item counts, and rescaled item-level values—is provided in Supplementary Materials. The Knapp–Hartung adjustment was used to compute more accurate and conservative 95% confidence intervals for the pooled mean, as recommended for meta-analyses with fewer than 30 effect sizes. Analyses were conducted in MetaAnalysisOnline.com [49]. Heterogeneity was assessed with the Q statistic, τ^2^, and I^2^ indices. Forest and funnel plots were generated to visualize study-level variability and publication bias. The final dataset included 8572 participants across 10 international samples (English, Arabic, Chinese, Turkish, Urdu, and Croatian versions of the DSS).

### 2.4. Thematic Analysis Phase

To complement the quantitative bibliometric and meta-analytic analyses, a thematic synthesis was conducted using the same deduplicated corpus of 215 unique publications retained from the bibliometric phase (note: an earlier merged export contained 228 records prior to final deduplication; the authoritative figure throughout this review is n = 215). Of these, 173 records contained sufficient author and index keyword data for co-occurrence network analysis after filtering out records with no usable keyword metadata. Author and index keywords, titles, and abstracts were analysed to detect co-occurrence patterns using bibliometric mapping procedures in Bibliometrix [10]. A keyword co-occurrence network was constructed, and community detection was performed using a modularity-based clustering algorithm [50] to group related terms into coherent thematic clusters. The network was then manually curated to refine cluster boundaries and remove indexing artifacts (e.g., medical subject headings such as human or female) to ensure conceptual clarity. Manual curation of cluster boundaries was conducted by both authors independently, followed by consensus discussion to resolve disagreements. Excluded indexing artifacts included generic Medical Subject Headings (e.g., ‘human,’ ‘female,’ ‘adult’) and unrelated disciplinary terms (e.g., ‘coal dust,’ ‘measurement’ in the engineering sense) that appeared in the network due to non-psychosocial uses of the term ‘digital stress.’ No formal inter-rater reliability coefficient was computed, given the algorithmic basis of the initial clustering; curation decisions were recorded and are available upon request.

Following the clustering, each thematic group was qualitatively interpreted through inductive content analysis of representative publications within the cluster. Themes were labelled to capture the dominant conceptual focus and interrelations among constructs. This combined computational–interpretive approach follows established practices for thematic mapping in scientific literature [11,51]. Four interconnected themes were identified—conceptual ambiguity, contextual moderators, the digital transformation paradox, and digital well-being and coping—which together form a multidimensional conceptual framework of digital stress.

## 3. Results

### 3.1. Bibliometric and Scientometric Findings

The bibliometric dataset comprised 215 documents published between 1981 and 2025, retrieved from 159 distinct sources including journals, books, and conference proceedings. The annual growth rate of publications on digital stress was 9.35%, indicating steady and expanding scholarly attention to the topic. On average, documents in this corpus were 4.47 years old and received 8.34 citations per publication, suggesting both recency and moderate impact. The dataset included 685 contributing authors, with a mean collaboration rate of 4.17 co-authors per paper and 17.21% of publications involving international co-authorship, reflecting an increasingly globalized research landscape. Author participation was broad, though single-authored works (n = 24) remained comparatively rare. The document types were dominated by journal articles (n = 150), complemented by conference papers (n = 11), proceedings papers (n = 19), and a smaller number of books, chapters, and reviews. Collectively, these descriptive indicators depict a maturing and collaborative research field characterized by growing productivity and diversified publication outlets. Citation data reveal that earlier foundational studies (e.g., 1994, 2017) exhibit higher average citations per year (above 4.0), suggesting enduring conceptual influence, whereas more recent outputs (post-2021) display lower annual citation rates due to their recency but are expected to accumulate impact over time.

The annual publication trend shows a slow and fragmented emergence of digital stress research from the early 1980s to 2010, with only isolated outputs in that period. A modest but consistent rise began after 2012, coinciding with intensified scholarly interest in technostress, digitalization, and online well-being. A marked inflection point occurred in 2020, with publications more than quadrupling in subsequent years, reaching a peak of 51 documents in 2025. This steep trajectory reflects the accelerated digital transformation following the COVID-19 pandemic and the growing recognition of digital stress as a key psychosocial and organizational issue. Overall, the trend demonstrates a transition from sporadic conceptual explorations to a consolidated, fast-expanding research domain.

The geographical distribution of publications reveals that digital stress research is concentrated in a small number of leading countries. Germany accounts for the largest share (n = 171), followed by the United States (n = 92) and China (n = 78), jointly representing the core centres of scientific output in this field. A secondary cluster includes Norway, Austria, the United Kingdom, and Canada, each contributing between 17 and 44 documents, indicating substantial engagement from European and North American institutions. Emerging contributors such as Romania, India, and the Netherlands show growing participation, reflecting diffusion of the topic to diverse research contexts. Although several countries from Asia, the Middle East, and South America appear in the dataset, their outputs remain limited to one or two publications. This distribution underscores a strong Euro-American dominance with expanding but still uneven global participation in digital stress scholarship.

The analysis of author keywords demonstrates a clear conceptual concentration around digital stress (n = 84), which overwhelmingly dominates the field (Figure 3). The next most frequent terms—stress management (18), social media (14), stress scale (9), and mental health (8)—highlight the psychological and applied orientations of current research. Additional recurring terms such as qualitative study, controlled trial, digital stressors, and university students indicate diversification in both methodology and study populations. Collectively, these findings show that digital stress research has consolidated around measurement and intervention themes while expanding toward contextual and experiential domains, particularly mental health and higher education.

As shown in Figure 4, the visualization presents the most frequent bigrams—two-word keyword combinations—appearing across the dataset. Early terms such as stress echocardiography (median 1995) reflected a biomedical orientation that preceded the digital era. After 2020, the emergence of digital stress, stress management, and social media indicates a decisive conceptual shift toward psychosocial and technological themes. When extending the analysis to trigrams, the most frequent phrases were digital stress echocardiography (1995–1997), digital stress management (2020–2023), and DSS (2023–2024). This progression underscores the conceptual maturation of the field, showing how “digital stress” evolved from a peripheral term to the central construct linking physiological, psychological, and digital well-being research.

As shown in Figure 5, the co-word network visualizes conceptual clusters identified through keyword co-occurrence. The central node digital stress shows the highest betweenness (511.16) and PageRank (0.21), confirming its position as the main integrative construct. Closely linked nodes such as technostress, social media, digitalization, and work stress form the dominant thematic nucleus, capturing the overlap between digital work environments, social interaction, and psychological strain. The coloured clusters reflect distinct thematic orientations: the orange cluster centres on psychological and organizational concepts (stress, mental health, well-being, anxiety); the blue and green clusters group health technology themes (digital health, mHealth, eHealth, stress management); the pink cluster represents applied and preventive domains (digitization, preventive measures, medical staff); and the brown cluster captures a smaller but distinct line of research related to engineering and material sciences, including terms such as measurement and coal dust. Collectively, these clusters indicate that while digital stress research is primarily psychological and technological, the term has also appeared in technical disciplines, reflecting its multidimensional and cross-domain relevance. 

As shown in Figure 6, the thematic evolution map traces the conceptual transition of digital stress research from 1981 to 2025. Early themes (1981–2023) such as stress, health, digitization, and mHealth provided the foundational structure of the field. Over time, these topics evolved toward contemporary clusters dominated by digital stress (2024–2025), social media, and digital health. The strong continuity between digital stress (1981–2023) and digital stress (2024–2025) indicates conceptual stability, while emerging links to digitalization and online vigilance suggest expansion into new digital behaviour contexts. The inclusion of artificial intelligence in the recent phase reflects a growing integration of digital stress studies with intelligent technologies and adaptive systems. This evolution highlights a gradual shift from general stress and technology use toward more complex frameworks involving digital ecosystems, social connectivity, and cognitive responses to technological environments.

### 3.2. Meta-Analytic Results

**Figure 7:**
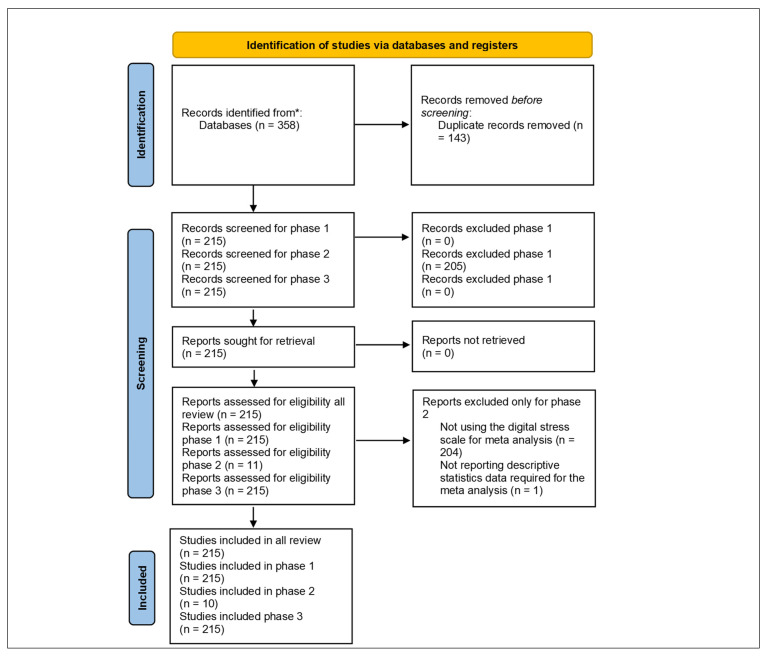
PRISMA 2020 Flow Diagram of Study Selection Process. * Scopus and Web of Science databases. *Note*. Phase 1: bibliometrics and scientometric analyses, phase 2: meta-analysis, and phase 3: thematic analysis.

Figure 7 presents the Preferred Reporting Items for Systematic Reviews and Meta-Analyses (PRISMA) 2020 flow diagram summarizing the identification, screening, and inclusion of records in this review [52]. The search process initially retrieved 358 records from Scopus and WoS, after which 143 duplicates were removed. The remaining 215 unique publications were screened for relevance to digital stress and related constructs. Following full-text assessment, 215 studies were retained for final bibliometric, scientometric, and thematic analyses, while eleven empirical studies using the DSS were identified, of which ten provided sufficient descriptive data for inclusion in the meta-analysis.

**Figure 8:**
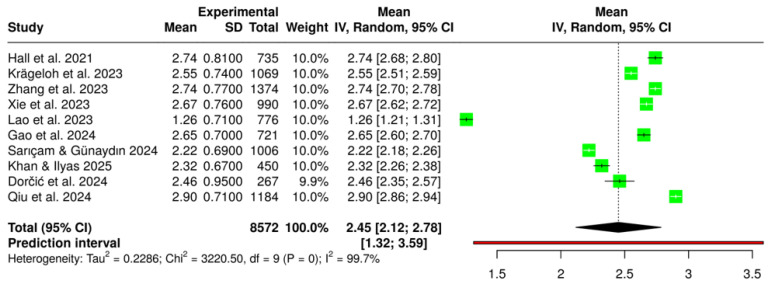
Forest Plot of Pooled Mean Effect of Digital Stress Levels Across Studies (Random-Effects Meta-Analysis). Studies included in meta-analysis: Hall et al. [1]; Krägeloh et al. [53]; Zhang et al. [54]; Xie et al. [55]; Lao et al. [56]; Gao et al. [57]; Sarıçam & Günaydın [58]; Khan & Ilyas [59]; Dorčić et al. [60]; Qiu et al. [18]. *Note.* Green squares represent individual study means, with square size proportional to study weight; horizontal lines indicate 95% confidence intervals. Squares positioned to the left of the dashed line indicate studies with below-average digital stress levels, squares to the right indicate above-average levels, and squares close to the dashed line indicate levels consistent with the pooled mean. The black diamond reflects the pooled mean with its 95% CI. The vertical dashed line marks the pooled mean. The red horizontal line indicates the 95% prediction interval.

After rescaling all DSS means to a 1–5 item-level metric, the pooled random-effects estimate indicated a mean digital stress level of 2.45 (95% CI = 2.12–2.78; τ^2^ = 0.23; I^2^ = 99.7%) (See Figure 8). The Knapp–Hartung adjustment slightly widened the confidence interval, providing a conservative estimate of the overall mean. Although heterogeneity was statistically significant (Q = 3220.5; *p* < 0.001), the variability may reflect contextual differences across cultures and scale versions. For reference purposes only, comparison with Hall et al. [1] U.S. normative sample places the international pooled mean at approximately the 36th percentile (‘average’ range, 26th–75th percentile). Given the substantial cultural heterogeneity (I^2^ = 99.7%), this comparison should be interpreted as a descriptive reference point against the scale’s original validation sample rather than as a normative benchmark for international populations. The 95% prediction interval (PI: 1.32–3.59) indicates that in a new study drawn from the same population of contexts, individual mean scores could range from low to moderately high, reflecting the substantial real-world variability underlying the pooled estimate. The high heterogeneity suggests substantial cross-context variability; formal moderator testing is warranted in future meta-analyses with larger study sets. Descriptively, student samples (k = 6) tended toward slightly lower means than employee or mixed samples (k = 4), consistent with contextual moderation hypotheses. Supplementary Materials contains summary statistics, heterogeneity statistics, and results from the test of heterogeneity. Supplementary Materials includes the characteristics of the studies and other data files related to this review.

**Figure 9:**
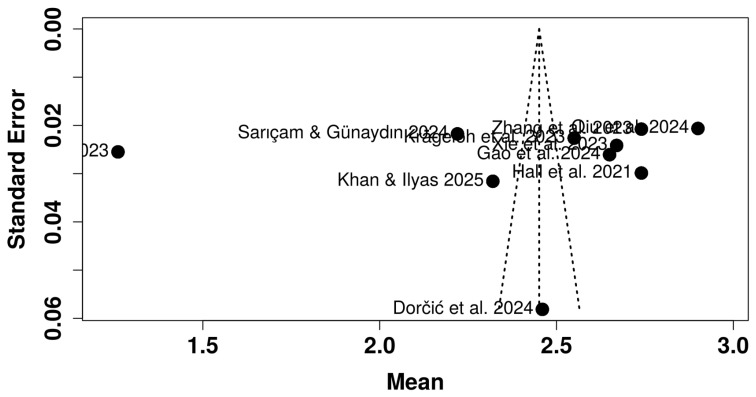
Funnel Plot for Assessing Publication Bias in Digital Stress Meta-Analysis. Studies included in meta-analysis: Hall et al. [1]; Krägeloh et al. [53]; Zhang et al. [54]; Xie et al. [55]; Lao et al. [56]; Gao et al. [57]; Sarıçam & Günaydın [58]; Khan & Ilyas [59]; Dorčić et al. [60]; Qiu et al. [18].

To contextualize the pooled value, comparison with Hall et al. [1] U.S. normative sample (M = 2.74, SD = 0.81) revealed that the international pooled mean corresponds to a z-score of −0.36, approximately the 36th percentile. According to Hall’s percentile categories, this places the pooled mean within the “average” range of digital stress (26th–75th percentile). Thus, across global samples, individuals tend to experience moderate levels of digital stress, slightly below those observed in the original English-speaking validation study. The funnel plot (Figure 9) appeared symmetrical, suggesting minimal publication bias, and the forest plot confirmed that most studies clustered between means of 2.2 and 2.9, indicating good cross-cultural consistency of the DSS total scores. Formal statistical tests for publication bias (e.g., Egger’s regression) are generally considered unreliable with fewer than 10–15 studies and were therefore not conducted. The funnel plot should be interpreted as a descriptive indicator only; the possibility of publication bias cannot be formally excluded, given the small number of included studies.

### 3.3. Thematic Synthesis

Before presenting the thematic clusters in detail, it is instructive to note the cross-methodological convergences and divergences observed across the three analytical phases. Bibliometrically, the sharp post-2020 publication surge and the dominance of ‘social media’ and ‘mental health’ as co-occurring keywords align with the thematic finding that contextual moderators—particularly academic and youth settings—have become central research priorities. The meta-analytic pooled mean of 2.45 (moderate stress level) is consistent with the thematic observation that digital stress is a normative psychosocial experience rather than a clinical threshold phenomenon, supporting the ‘digital well-being and coping’ cluster’s framing of digital stress as a manageable rather than pathological condition. However, a divergence is notable: while the meta-analysis found high heterogeneity (I^2^ = 99.7%), suggesting strong moderating effects of cultural and contextual factors, the bibliometric dataset remains disproportionately Euro-American in origin—a structural gap that the thematic synthesis of contextual moderators confirms as a priority for future research. Together, these three analytical perspectives offer a triangulated and mutually reinforcing account of digital stress as a distinct, context-sensitive, and increasingly well-characterized research domain.

**Figure 10:**
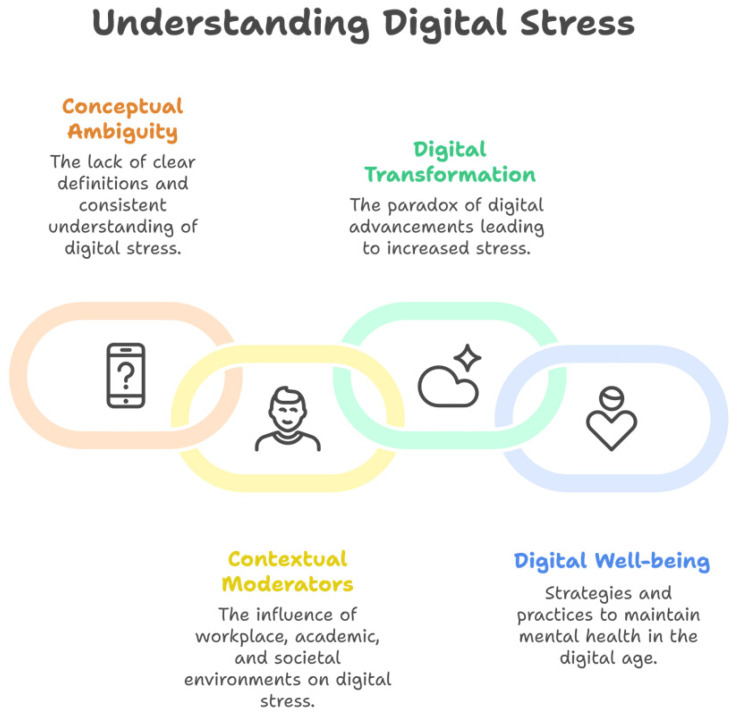
Integrative Conceptual Framework of Digital Stress. *Note.* This figure is a purely illustrative conceptual representation of the four thematic dimensions derived from the thematic synthesis. It is not a formal model or statistical output. It was generated using https://www.napkin.ai/ (accessed on 10 January 2026) based on the four cluster labels and their descriptive summaries. This figure synthesizes the convergent findings of the bibliometric, meta-analytic, and thematic analytical phases, representing the four interconnected dimensions that collectively define the construct’s theoretical boundaries and research landscape. *Note*: An integrative representation of the four thematic dimensions derived from the literature: conceptual ambiguity, contextual moderators, the paradox of digital transformation, and digital well-being. The figure was generated using https://www.napkin.ai/ (accessed on 10 January 2026).

To complement the bibliometric and meta-analytic analyses, a thematic synthesis was conducted to interpret the conceptual evolution of digital stress research. Author and index keywords from the merged Scopus–WoS dataset (n = 215 unique records) were analysed using keyword co-occurrence and community detection, followed by manual curation of conceptually coherent clusters. The resulting network (n = 173 digital-stress–related papers) revealed four dominant conceptual themes: (1) conceptual ambiguity and definitional drift; (2) contextual moderators across workplace, academic, and societal settings; (3) the digital transformation paradox; and (4) digital well-being and coping strategies. Each theme reflects a distinct yet interconnected research stream that collectively captures the multidimensional nature of digital stress. Figure 10 illustrates the four interrelated conceptual dimensions identified through the thematic synthesis. The visualization highlights how digital stress is not a single construct, but an interconnected phenomenon shaped by definitional ambiguity, contextual influences, paradoxes of digital transformation, and evolving approaches to digital well-being. These dimensions are conceptually linked, indicating that progress in addressing digital stress requires simultaneous attention to measurement clarity, socio-technical contexts, systemic digital change, and individual or organizational coping strategies.

**Conceptual Ambiguity and Definitional Drift.** In this study, a systematic search across WoS and Scopus was conducted to trace the historical development and disciplinary migration of the term “digital stress.” The earliest indexed record in WoS dates back to 1981, though it was excluded due to the lack of an abstract and accessibility, while the earliest retrievable study in Scopus appeared in 1987. To construct a coherent trajectory of meaning, the earliest accessible and thematically relevant works from each discipline were selected up to 2016, marking the period when digital stress began to acquire explicit psychological and emotional dimensions. As summarized in Table 1, the concept initially appeared in biomedical and engineering contexts, describing physically measurable forms of stress—such as cardiac load, electrical imbalance, or material strain—before migrating into psychosocial and communicative frameworks. This shift illustrates a definitional transformation from digitally measured stress in technical systems to stress experienced through digital contexts in human environments, culminating in contemporary perspectives that integrate emotional, behavioural, and social facets of technological engagement.

Representative keywords and papers anchoring each theme are as follows: (1) Conceptual ambiguity—‘technostress,’ ‘digital overload,’ ‘online vigilance’ [1,7]; (2) Contextual moderators—‘workplace,’ ‘university students,’ ‘autonomy’ [2,16]; (3) Digital transformation paradox—‘digitalization,’ ‘burnout,’ ‘work stress’ [3,61]; (4) Digital well-being and coping—‘mindfulness,’ ‘digital hygiene,’ ‘recovery’ [31,62].

**Table 1:** Historical and Disciplinary Development of the Concept “Digital Stress” (1987–2016).

In-Text Citation	Research Focus	Definition of Digital Stress	Measurement	Disciplinary Lens
[63]	Detecting ischemia via mitral flow velocity during exercise	Digitally recorded cardiac stress testing using echocardiography	Doppler ultrasound during treadmill exercise	Medical imaging/Cardiology
[64]	Load balancing in paralleled switching converters	Digitally communicated electrical load sharing among converters	Design and testing of single-wire protocol	Electrical engineering/Power systems
[65]	Measuring internal stress in KDP crystals	Digitally measured birefringence and optical axis deviation	High-precision optical instrumentation	Optics/Materials Science
[66]	Hypothesizing health risks from electromagnetic exposure	Chronic neurological strain from electrified environments	Theoretical overview and hypothesis	Environmental Neuroscience/Toxicology
[67]	Designing a system to induce and regulate stress digitally	Psychophysiological stress state induced and modulated via digital feedback loops	Development of GASICA system with sensors and feedback algorithms	Human–Computer Interaction/Neuroinformatics
[68]	Mapping geological stress for gas reservoir exploration	Digitally visualized subsurface stress patterns	Integration of well data and visualization software	Geomechanics/Petroleum Engineering
[69]	Reducing voltage drift in bandgap references due to mechanical stress	Digitally sensed and compensated mechanical strain on silicon chips	On-chip stress sensor; thermal cycling; compensation algorithm	Semiconductor physics/Sensor design
[8]	Emotional and relational stress in digital environments	Emotional/social strain from online interactions and connectivity pressure	Thematic analysis of 2000 anonymous posts	Psychology/Adolescent development
[9]	Exploring how digital media environments contribute to chronic stress	Cognitive and emotional overload from constant connectivity and multitasking	Theoretical synthesis in a book chapter	Media Psychology/Communication Studies

Note. All records in this table were retrieved by the bibliometric search and are included in the full corpus of 215 documents. They are presented here illustratively to trace the construct’s disciplinary migration; non-psychosocial records are retained in the bibliometric counts but excluded from the thematic and meta-analytic phases (see Section 1.1 scope statement).

**Figure 11:**
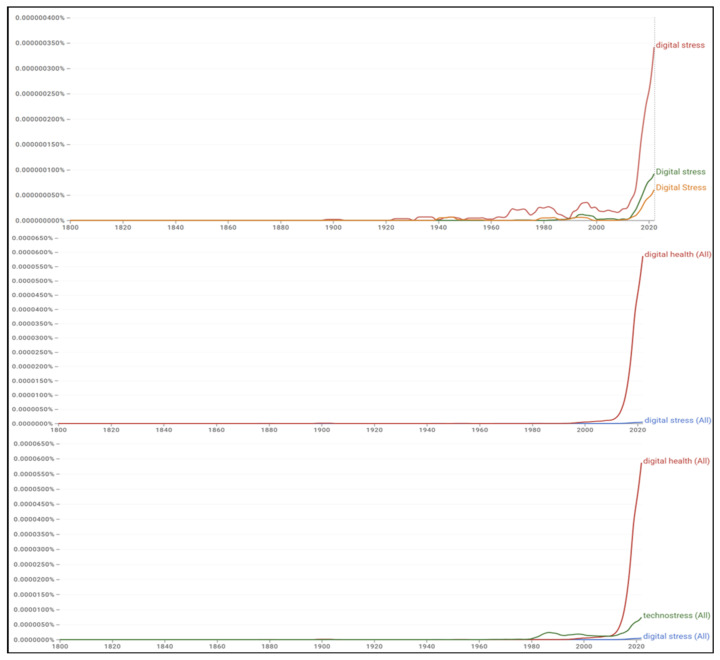
Google Books Ngram Trends for “Digital Stress,” “Digital Health,” and “Technostress” (1800–2019).

As shown in Figure 11, occurrences of the phrase “digital stress” are negligible until the 2000s and then rise steeply after 2015, with a sharp post-2020 uptick in the current dataset. “Digital health” shows an earlier and much larger surge beginning around 2010 and remains orders of magnitude more frequent, indicating that the broader health–technology discourse has dominated book usage relative to the more specific construct of digital stress. “Technostress” appears earlier than digital stress, with small waves in the 1990s and 2000s, but is eclipsed by digital health and, more recently, by digital stress.

Note: The Ngram Viewer plots normalized relative frequencies, so the y-axis represents the proportion of all unigrams or n-grams in the selected corpus; lines labeled “(All)” reflect case-insensitive aggregates. Because Google periodically updates the corpus and OCR, trend lines can shift with new releases, including the July 2024 dataset update and other corrections [70,71].

Even within the cognitive and emotional use of the concept, the main aim of this study, it still reflects the persistent conceptual turbulence surrounding “digital stress.” Across the literature, researchers employ overlapping constructs such as technostress, online vigilance, and digital overload, often interchangeably but with distinct theoretical underpinnings. Early frameworks emphasized exposure to technology-induced demands as a source of cognitive strain [7], whereas more recent approaches conceptualize digital stress as an affective, context-dependent experience shaped by social comparison, connectivity, and media use patterns [1]. Psychometric contributions (e.g., [5,72] demonstrate a continuing effort toward definitional refinement through the development and validation of measurement tools that balance conceptual precision with ecological validity. This ongoing definitional drift underscores a field in active negotiation, oscillating between mechanistic, cognitive, and experiential interpretations of digital stress.

**Contextual Moderators: Workplace, Academic, and Societal.** A major thread emphasizes where and for whom digital stress occurs. Organizational and educational contexts dominate, revealing structural determinants such as digital workloads, autonomy, managerial support, and digital literacy. Studies of employees [2] and healthcare professionals [4] highlight that job demands–resources configurations moderate the relationship between technology exposure and strain. In academic and youth settings, continuous connectivity, performance pressure, and online presence anxiety emerge as critical moderators. This theme captures the translation of digital stress from an individual psychological issue into a socio-technical and institutional concern.

**The Digital Transformation Paradox.** The “digital stress paradox” encapsulates a central dilemma: while digital transformation promises flexibility and efficiency, it simultaneously introduces information overload, constant availability, and technostress or proliferation. Empirical studies [3,61] demonstrate that the same systems designed to optimize productivity often exacerbate cognitive strain and emotional exhaustion. This duality echoes the paradox of technology acceptance—innovation and burden coexisting. Thematic patterns indicate that successful digital transformation requires not only technical adaptation but also psychosocial redesign of work routines and boundary management norms.

**Digital Well-Being and Coping Strategies.** Recent literature marks a conceptual shift toward resilience, coping, and digital well-being. Research explores both individual and organizational strategies: self-regulation, mindfulness, digital hygiene, structured breaks, and policies limiting after-hours connectivity. Scholars emphasize positive digital engagement—leveraging technology for recovery rather than exhaustion [31,62]. The emerging narrative reframes digital stress from an inevitable side-effect of connectivity to a manageable psychosocial process contingent on literacy, boundaries, and cultural norms. This theme aligns with the wider move toward digital well-being and sustainable human–technology interaction.

## 4. Discussion

The triangulated results from bibliometric, scientometric, meta-analytic, and thematic analyses collectively demonstrate that digital stress has evolved from a loosely used label into a distinct, maturing research construct that bridges technological, psychological, and organizational domains. However, the conceptual identity of digital stress remains partially fluid, oscillating between being a successor to technostress and a broader framework encompassing socio-digital demands. The bibliometric trajectory confirmed a rapid expansion of the field after 2020, largely driven by pandemic-induced digital transformation and the mainstreaming of digital well-being discourse [2,34]. Yet, keyword co-occurrence and thematic clustering revealed persistent conceptual overlap with technostress, online vigilance, and digital overload, illustrating that theoretical consolidation is still underway. This definitional turbulence echoes earlier phases in stress research where constructs proliferated before integrative frameworks stabilized [7,8]. The increasing use of frameworks such as the Job Demands–Resources model and the Transactional Model of Stress [32,38] suggests a gradual theoretical maturation, as these models collectively explain how digital demands become stressors and how cognitive appraisals and coping determine individual outcomes. Empirical support for this integration is visible across workplace and academic studies showing that digital workloads and connectivity pressures predict emotional exhaustion, moderated by job autonomy and social support [24,25,36]. Indicators of this theoretical maturation, as observable in the present mapping outputs, include: the convergence of publication growth after 2020, the stabilization of ‘digital stress’ as the dominant keyword in the co-occurrence network, the increasing citation of JD-R and transactional stress frameworks across included studies, and the cross-linguistic replication of the DSS factor structure across six language versions. In brief, digital stress is distinguished from technostress by its social-evaluative dimension (linking to the conceptual ambiguity theme), its cross-contextual scope (contextual moderators theme), its paradoxical relationship with digital affordances (digital transformation paradox theme), and its orientation toward recovery and boundary management (digital well-being theme).

The meta-analytic synthesis of DSS studies strengthens this conceptual distinctiveness by confirming consistent psychometric performance across linguistic and cultural adaptations, with a pooled mean reflecting moderate digital stress levels worldwide. These convergent results affirm that, despite contextual variability, digital stress manifests with comparable intensity across diverse populations [1,5,27]. Nonetheless, the high heterogeneity (I^2^ = 99.7%) indicates that structural and cultural moderators meaningfully shape digital stress experiences—a finding that parallels contextual disparities observed in other psychosocial domains such as depression and anxiety [73]. Just as national epidemiological patterns reveal uneven symptom distributions due to systemic and cultural factors, digital stress likewise reflects sociotechnical inequalities in digital literacy, access, and coping resources. The thematic synthesis further clarifies that this heterogeneity aligns with definitional ambiguity and contextual moderators: in work settings, digital transformation paradoxes dominate [3], whereas in youth and academic contexts, social comparison and fear of missing out serve as primary stress pathways [16,19,60]. Across both domains, digital stress operates not merely as a reaction to technological exposure but as an indicator of broader psychosocial adaptation challenges within increasingly mediated environments. A dedicated psychometric meta-analysis pooling reliability coefficients (α/ω) and factor-analytic fit indices across DSS validation studies would constitute a valuable and distinct future contribution, extending beyond the level-consistency focus of the present synthesis. In this broader context, digital interventions are also increasingly recognized as tools for psychological rehabilitation and well-being promotion, reinforcing the dual role of technology as both stressor and resource [74]. 

The three analytical strands converge on a coherent picture of digital stress as a maturing, context-sensitive construct. Bibliometrically, the field has undergone rapid growth and conceptual specialization since 2020, with ‘digital stress’ consolidating as a distinct node in the keyword network. Meta-analytically, DSS mean scores are comparably moderate across six language communities, suggesting that the construct manifests with broadly similar intensity globally, even as high heterogeneity (I^2^ = 99.7%) confirms the role of cultural and contextual moderators. Thematically, the four identified dimensions—conceptual ambiguity, contextual moderators, the digital transformation paradox, and digital well-being—map onto precisely those moderating factors: ambiguity sustains heterogeneity; contextual variation drives it; the transformation paradox explains its post-2020 surge; and the well-being focus reflects the field’s applied response. Figure 10 synthesizes these converging insights into an integrative conceptual framework of digital stress.

Taken together, and within the scope of the bibliometric, meta-analytic, and thematic methods employed, the present findings are consistent with positioning digital stress as a multidimensional construct at the intersection of technological change and human well-being. Caution is warranted, however, in generalizing beyond the construct-level and psychometric insights yielded by this review, given the cross-sectional and self-report predominance of the included studies. Its theoretical consolidation appears to be progressing through the integration of structural models (e.g., Job Demands–Resources model) with process-oriented frameworks (e.g., Transactional Model), and through the refinement of measurement approaches that now balance subjective self-reports with objective and physiological indicators [13,43,45]. The field’s next conceptual frontier lies in connecting digital stress research to broader models of emotional regulation and public mental health, thereby linking everyday digital strain to systemic well-being outcomes in line with global mental health priorities [73].

### Strengths and Limitations

A key strength of this study lies in its multi-method design integrating bibliometric, scientometric, and meta-analytic evidence, allowing both structural and quantitative validation of trends in digital stress research. The inclusion of recent large-scale validations of the DSS adds empirical robustness and psychometric clarity to an evolving domain.

However, several limitations must be acknowledged. The field’s conceptual heterogeneity constrained meta-analytic precision, and the cross-sectional predominance of included studies limits causal inference. Publication bias toward English-language research may underrepresent culturally diverse experiences of digital stress, and the integration of physiological and behavioural indicators remains limited, with the evidence base relying heavily on self-report data. The bibliometric corpus is anchored on the literal phrase ‘digital stress,’ which may under-sample synonym-based literature and inflate non-psychosocial contributions to country output and keyword counts; a sensitivity analysis restricting the corpus to psychosocial records is recommended for future work. The high heterogeneity (I^2^ = 99.7%) and small number of studies (k = 10) preclude formal subgroup meta-regression; contextual moderation should therefore be treated as a theoretically informed interpretation rather than an empirically confirmed finding, and future DSS meta-analyses with larger study sets are encouraged to test moderators including language version, population type, and data collection year. Finally, pooling means across DSS versions with 17–31 items introduces comparability assumptions, and the wide prediction interval (1.32–3.59) confirms that the pooled mean of 2.45 reflects broad cross-cultural variability rather than a stable global normative value.

## 5. Conclusions

In sum, this review establishes digital stress as a distinct yet conceptually connected construct within the broader technostress and digital well-being literature. To our knowledge, this is the first review to integrate construct-specific bibliometric and scientometric mapping, Google Ngram trend analysis, a random-effects meta-analysis of DSS mean scores, and a keyword co-occurrence thematic synthesis specifically for the labeled construct ‘digital stress’—distinguishing it from adjacent reviews focused on technostress or digital workplace well-being more broadly. By combining structural mapping, theoretical synthesis, and quantitative aggregation, this study demonstrates that digital stress reflects multidimensional pressures stemming from connectivity demands, social expectations, and continuous digital engagement. The evidence indicates that digital stress contributes to both psychological strain and adaptive behaviours, depending on contextual and individual moderators. The findings consolidate the construct’s theoretical boundaries and measurement standards while highlighting the need for longitudinal, cross-cultural, and multimodal research designs. Digital stress thus emerges as a critical focus for understanding human adaptation in technology-saturated environments, linking cognitive, emotional, and systemic determinants of well-being in the digital age. Integrating across all three analytical lenses, this review yields three overarching generalizations. First, digital stress has completed a definitional transition from a technical engineering term to a psychosocial construct with its own measurement infrastructure and theoretical grounding—a transition traceable through both the Ngram and bibliometric data. Second, the construct’s cross-cultural stability at the level of mean scores, combined with substantial contextual heterogeneity, indicates that digital stress is simultaneously universal in its presence and particular in its expression—a duality that future intervention research must address. Third, the digital transformation paradox identified thematically represents perhaps the most consequential practical insight: the same technologies intended to enhance productivity and connection are primary sources of the strain this review documents. Addressing digital stress therefore requires not only individual coping strategies but systemic redesign of digital environments, organizational policies, and educational frameworks for sustainable human–technology interaction.

## Data Availability

The bibliometric dataset (search strings, export date, deduplication rules, and cleaned record list), the extracted meta-analysis table (study identifiers, reported and rescaled means, SDs, sample sizes, item counts), and the keyword co-occurrence adjacency matrix are provided as Supplementary Materials. No additional external data are available.

## Supplementary Materials

The following supporting information can be downloaded at: https://www.mdpi.com/article/10.3390/healthcare14060823/s1.
