# Supplementary material for: Digital Stress: Insights from Bibliometric, Scientometric, Meta-Analytic and Thematic Analyses
Source: Healthcare (Basel). 2026 Mar 23;14(6):823. doi: 10.3390/healthcare14060823 (PMC13027238; doi:10.3390/healthcare14060823)
Supplement: Supplementary file 1 [file healthcare-14-00823-s001.zip › Supplementary File S2. SearchStrings.pdf]

Supplementary File S2

Complete Search Strings Across All Databases

Search date: 31 October 2025. No language restrictions applied. All document types included.

#	Database	Search String	Field	Results
1	Scopus	`TITLE("Digital Stress")`	Title	80
2	Scopus	`TITLE-ABS-KEY("Digital Stress")`	Title / Abs / Key	194
3	Scopus	`TITLE-ABS-KEY("Digital Stress Scale")`	Title / Abs / Key	11
4	Scopus	`TITLE-ABS-KEY("multidimensional digital stress scale")`	Title / Abs / Key	4
5	Scopus	`(TITLE-ABS-KEY("Digital Stress Scale") AND TITLE-ABS-KEY("DSS"))`	Title / Abs / Key	7
6	Scopus	`(TITLE("Digital Stress Scale") AND TITLE("DSS"))`	Title	1
7	Web of Science	`"digital stress" (Topic)`	Topic (TS)	164
8	Web of Science	`"digital stress" (Title)`	Title (TI)	73
9	Web of Science	`"digital stress scale" (All Fields)`	All Fields	9
10	Web of Science	`"digital stress scale" (Topic)`	Topic (TS)	9
11	Web of Science	`"digital stress scale" (Title)`	Title (TI)	7
12	Web of Science	`"multidimensional digital stress scale" (Title)`	Title (TI)	1
13	Web of Science	`"multidimensional digital stress* scale" (Title)`	Title (TI)	1
		**Scopus subtotal (before deduplication)**		**194**
		**Web of Science subtotal (before deduplication)**		**164**
		**Combined total after merging and deduplication**		**215**

Notes.

Scopus searches were conducted via the Scopus Advanced Search interface. Web of Science searches were conducted via the WoS Core Collection. Results counts reflect records retrieved on 31 October 2025 and may differ from current database totals. Scopus yielded 194 unique records and Web of Science yielded 164; after merging and deduplication using the bibliometrix R package, 215 unique records were retained. These 215 records were used for the bibliometric, scientometric, and thematic analyses. Of these, 11 empirical studies employed the Digital Stress Scale (DSS); 10 provided sufficient descriptive statistics and were included in the meta-analysis.
