# Supplementary material for: Digital Stress: Insights from Bibliometric, Scientometric, Meta-Analytic and Thematic Analyses
Source: Healthcare (Basel). 2026 Mar 23;14(6):823. doi: 10.3390/healthcare14060823 (PMC13027238; doi:10.3390/healthcare14060823)
Supplement: Supplementary file 1 [file healthcare-14-00823-s001.zip › Supplementary File S1 for the Meta Analysis Phase.pdf]

Supplementary File S1 for the Meta Analysis Phase

A

Summary Tables

Study	N	Mean	SD	95% CI (Lower–Upper)	Weight (%)
Hall et al., 2021	735	2.74	0.81	2.68 – 2.80	10.00
Krägeloh et al., 2023	1069	2.55	0.74	2.51 – 2.59	10.02
Zhang et al., 2023	1374	2.74	0.77	2.70 – 2.78	10.02
Xie et al., 2023	990	2.67	0.76	2.62 – 2.72	10.01
Lao et al., 2023	776	1.26	0.71	1.21 – 1.31	10.01
Gao et al., 2024	721	2.65	0.70	2.60 – 2.70	10.01
Sarıçam & Günaydın, 2024	1006	2.22	0.69	2.18 – 2.26	10.02
Khan & Ilyas, 2025	450	2.32	0.67	2.26 – 2.38	10.00
Dorčić et al., 2024	267	2.46	0.95	2.35 – 2.57	9.89
Qiu et al., 2024	1184	2.90	0.71	2.86 – 2.94	10.02
Random effects model	8572	2.45	0.75	2.12 – 2.78	100.00

B

Quantifying Heterogeneity

Parameter	Value	95% CI
τ^2	0.23	[0.105 – 0.777]
τ	0.48	[0.324 – 0.881]
I^2	1.00	[0.997 – 0.998]
H	18.92	[17.54 – 20.40]

C

Test of Heterogeneity

Q	df	p-value
3220.50	9	0.00

D

Historical and Disciplinary Development of the Concept “Digital Stress” (1987–2016)

In-Text Citation	Research Focus	Definition of Digital Stress	Measurement	Disciplinary Lens
(Mitchell et al., 1988)	Detecting ischemia via mitral flow velocity during exercise	Digitally recorded cardiac stress testing using echocardiography	Doppler ultrasound during treadmill exercise	Medical imaging / Cardiology
(Rinne et al., 2010)	Load balancing in paralleled switching converters	Digitally communicated electrical load sharing among converters	Design and testing of single-wire protocol	Electrical engineering / Power systems
(Wei et al., 2011)	Measuring internal stress in KDP crystals	Digitally measured birefringence and optical axis deviation	High-precision optical instrumentation	Optics / Materials Science

(Manzetti & Johansson, 2012)	Hypothesizing health risks from electromagnetic exposure	Chronic neurological strain from electrified environments	Theoretical overview and hypothesis	Environmental Neuroscience / Toxicology
(van der Vijgh et al., 2014)	Designing a system to induce and regulate stress digitally	Psychophysiological stress state induced and modulated via digital feedback loops	Development of GASICA system with sensors and feedback algorithms	Human-Computer Interaction / Neuroinformatics
(Perumalla et al., 2014)	Mapping geological stress for gas reservoir exploration	Digitally visualized subsurface stress patterns	Integration of well data and visualization software	Geomechanics / Petroleum Engineering
(Motz et al., 2015)	Reducing voltage drift in bandgap references due to mechanical stress	Digitally sensed and compensated mechanical strain on silicon chips	On-chip stress sensor; thermal cycling; compensation algorithm	Semiconductor physics / Sensor design
(Weinstein & Selman, 2016)	Emotional and relational stress in digital environments	Emotional/social strain from online interactions and connectivity pressure	Thematic analysis of 2000 anonymous posts	Psychology / Adolescent development
(Hefner & Vorderer, 2016)	Exploring how digital media environments contribute to chronic stress	Cognitive and emotional overload from constant connectivity and multitasking	Theoretical synthesis in a book chapter	Media Psychology / Communication Studies
